# A Peculiar Case

**Published:** 1888-12

**Authors:** Alice B. Campbell

**Affiliations:** Brooklyn


### A PECULIAR CASE.

ALICE B. CAMPBELL, M. D., BROOKLYN.

Mr. G., aged sixty, very spare and lean, maker of complicated watches. November 20th, Sunday, was suddenly seized after dinner with vomiting, accompanied with inability to talk. Attack passed off and he seemed well.

Following morning, after breakfast, taken with same symptoms more violently, vomiting any food or drink taken. The smell of what was thrown up and from mouth was very offensive, resembling decayed shell-fish. The expression of his face was one of anguish and anxiety—wrinkled and very old. He was unable to put either words or ideas together; could only reply in detached words. He was restless, and from what I could gather of his utterances, he felt worse lying down. Thirsty, but water did not feel good in his stomach. Moaning and lamenting, saying, "Mind gone." I had him go to bed, where he tossed all that day and following night. Gave Ars.

Next morning, Tuesday, found no change with the exception of the vomiting, which had ceased, and he was able to retain liquid food, and an additional symptom, that of constant spitting, which had come on in the night, and was now, in the morning, being continued. Spitting every minute, anywhere, regardless of persons or things. Evening found him in the same condition. Indifferent to outside influences. Mental condition ever worse. Does not reply to questions, and did not know his own brother. At eight P. M. was seized with total blindness. Called upon him at midnight, found pupils nearly insensible to artificial light. He got one dose of medicine Phos. at half-past twelve A. M. Went to sleep at one o'clock for the first since attack, and slept till five A. M. Awoke with his sight, and better in every respect ; less restless ; would answer questions ; asked for music on organ, and could tell the names of the tunes. He still kept up the spitting, and could only talk in a slow, detached, and unfinished manner. The sight of a watch seemed to rouse him as nothing else did, but he could not tell time. Had no desire for anything.

Next day, Thursday, the spitting was better, and there was a general improvement.

The next day, Friday, his first remark was that he could tell time, and remembered trying the day before. After a little study he gave the correct time.

Next day, Saturday, he was up, dressed, and said aside from weakness he felt as well as he ever did. The following Wednesday he called at my office, and a week afterward returned to his business.

At beginning of attack pulse about ninety, and rather small. After vomiting ceased it fell below normal, but became fuller.

